# Mechanisms of PANoptosis and relevant small-molecule compounds for fighting diseases

**DOI:** 10.1038/s41419-023-06370-2

**Published:** 2023-12-21

**Authors:** Lian Wang, Yanghui Zhu, Lu Zhang, Linghong Guo, Xiaoyun Wang, Zhaoping Pan, Xian Jiang, Fengbo Wu, Gu He

**Affiliations:** 1 Department of Dermatology & Venerology and Department of Pharmacy, West China Hospital, Sichuan University, Chengdu, Sichuan 610041 P. R. China; 2 Laboratory of Dermatology, Clinical Institute of Inflammation and Immunology (CIII), Frontiers Science Center for Disease-related Molecular Network and State Key Laboratory of Biotherapy, West China Hospital, Sichuan University and Collaborative Innovation Center of Biotherapy, Chengdu, 610041 China

**Keywords:** Small molecules, Pharmacodynamics

## Abstract

Pyroptosis, apoptosis, and necroptosis are mainly programmed cell death (PCD) pathways for host defense and homeostasis. PANoptosis is a newly distinct inflammatory PCD pathway that is uniquely regulated by multifaceted PANoptosome complexes and highlights significant crosstalk and coordination among pyroptosis (P), apoptosis (A), and/or necroptosis(N). Although some studies have focused on the possible role of PANpoptosis in diseases, the pathogenesis of PANoptosis is complex and underestimated. Furthermore, the progress of PANoptosis and related agonists or inhibitors in disorders has not yet been thoroughly discussed. In this perspective, we provide perspectives on PANoptosome and PANoptosis in the context of diverse pathological conditions and human diseases. The treatment targeting on PANoptosis is also summarized. In conclusion, PANoptosis is involved in plenty of disorders including but not limited to microbial infections, cancers, acute lung injury/acute respiratory distress syndrome (ALI/ARDS), ischemia-reperfusion, and organic failure. PANoptosis seems to be a double-edged sword in diverse conditions, as PANoptosis induces a negative impact on treatment and prognosis in disorders like COVID-19 and ALI/ARDS, while PANoptosis provides host protection from HSV1 or *Francisella novicida* infection, and kills cancer cells and suppresses tumor growth in colorectal cancer, adrenocortical carcinoma, and other cancers. Compounds and endogenous molecules focused on PANoptosis are promising therapeutic strategies, which can act on PANoptosomes-associated members to regulate PANoptosis. More researches on PANoptosis are needed to better understand the pathology of human conditions and develop better treatment.

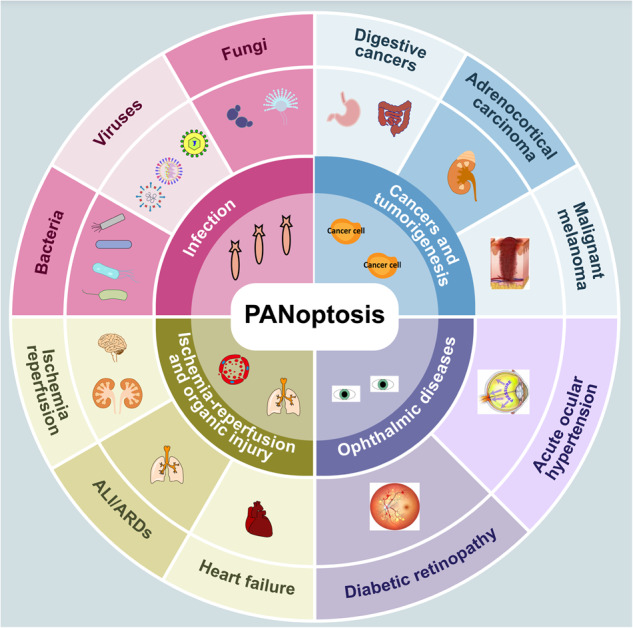

## Facts

1. PANoptosis is a novel and distinct inflammatory PCD pathway that is uniquely regulated by the multifaceted PANoptosome complexes.
2. The involvement of PANoptosis extends to various disorders, not limited to microbial infections, cancers, ALI, ARDS, ischemia-reperfusion, and organ failure.
3. Compounds and endogenous molecules that specifically target the molecules involved in the formation of PANoptosome hold promise for improving disease outcomes.

## Open questions

1. How do cells balance the regulatory mechanisms underlying PANoptosis engage in crosstalk?
2. What are the interactions between the PANoptosis pathway and those of other types of cell death subroutines?
3. Are small-molecule compounds that target PANoptosis suitable for use in clinical trials?

## Introduction

Programmed cell death (PCD), which has specific genetically encoded requirements, is a vital process during development and plays an important role in homeostasis and in host defense against different pathogens and stimuli [1]. Dysregulation of the progression of PCD is closely linked to different diseases, including infectious diseases, autoimmune diseases, and cancers. Although different PCD pathways have been identified, the most well-defined and classical PCD pathways are pyroptosis, apoptosis and necroptosis [2].

Pyroptosis, which is characterized by cell swelling, pore formation in the cell membrane, membrane rupture, and the release of cell contents, is a classical type of inflammatory PCD pathway [2]. In response to stimuli, gasdermin D (GSDMD) and other GSDM members can be cleaved by active caspases (e.g., caspase-1/3/4/5/8/11) or granzymes (e.g., granzyme A/B) and form large pores in the cell membrane to release cell contents, induce pyroptosis, and stimulate inflammation. Apoptosis, which is activated by extrinsic and intrinsic pathways, is a highly conserved physiological PCD pathway that is characterized by cell shrinkage, internucleosomal DNA fragmentation, nuclear condensation, and the formation of apoptotic bodies, while maintaining the integrity of cell membranes [3]. In response to drugs, hypoxia, high temperature, and other exogenous factors, proapoptotic death receptors (DRs), such as Fas, tumor necrosis factor receptor 1/2 (TNFR1/2), and DR4/5, can bind with specific ligands, trimerize and aggregate within the cell membrane, resulting in the recruitment of adaptor proteins, such as Fas-associated death domain proteins (FADDs) and caspase-8/10, and induce extrinsic apoptosis. In the intrinsic pathway, B-cell leukemia/lymphoma 2 (Bcl-2) and Bcl-2-associated X, apoptosis regulator (Bax) on the mitochondrial membrane can be activated by DNA damage, oxidative stress and other internal stimuli, which is followed by the release of cytochrome c, which links procaspase-9 and apoptotic protease activating factor 1 (APAF1) to form an apoptosome. The apoptosome cleaves procaspase-9 to caspase-9, which further activates and cleaves caspase-3/6/7 and then induces apoptosis [4]. Moreover, with the disruption of the cell membrane, swelling of cell bodies and organelles, and fragmented chromatin, necroptosis is regarded as a nonapoptotic form of PCD [3]. In brief, when caspase-8 is inhibited, receptor-interacting protein kinase 1 (RIPK1), RIPK3, mixed lineage kinase domain-like protein (MLKL), FADD, and procaspase-8 form complex IIb via the TNF or Toll-like receptor (TLR) pathway, which leads to necroptosis [4].

Historically, these PCD pathways have been regarded as pathways that are independent and separate from each other. However, recent studies have indicated significant and extensive crosstalk among these three pathways [1, 2]. Exploring the complex interplay among these PCD pathways provides insights into how these types of cell death are executed and regulated to control diseases. PANoptosis, which was newly termed by the Kanneganti team in 2019, is a distinctly inflammatory PCD pathway that integrates pivotal components from other cell death pathways, including pyroptosis (P), apoptosis (A), and/or necroptosis (N), highlighting their crosstalk and coordination [5]. Importantly, the integrity of biological functions during PANoptosis cannot be simply explained by pyroptosis, apoptosis, or necroptosis alone, and only blocking anyone form of these cell death pathways does not effectively prevent PANoptosis progression. Furthermore, PANoptosis is characterized by the involvement of critical cell death signaling factors associated with pyroptosis, apoptosis, and/or necroptosis, followed by the formation of a multifaceted scaffolding compound termed the PANoptosome [6]. Emerging evidence has shown that PANoptosis can be induced in different physiological conditions, such as viral and bacterial infections and cancers [7, 8]. The possible roles of PANoptosomes and PANoptosis in infectious diseases have been reviewed [9, 10]. To our knowledge, the role of PANoptosis and related agonists or inhibitors in disorders has not yet been thoroughly reviewed and discussed. In this review, we mainly focused on the contribution of PANoptosomes and PANoptosis to multiple pathological conditions and human diseases. The therapeutic potential of PANoptosis modulation and relevant small-molecule compounds for disease prevention and treatment was also discussed.

## PANoptosis and PANoptosome

**Fig. 1:**
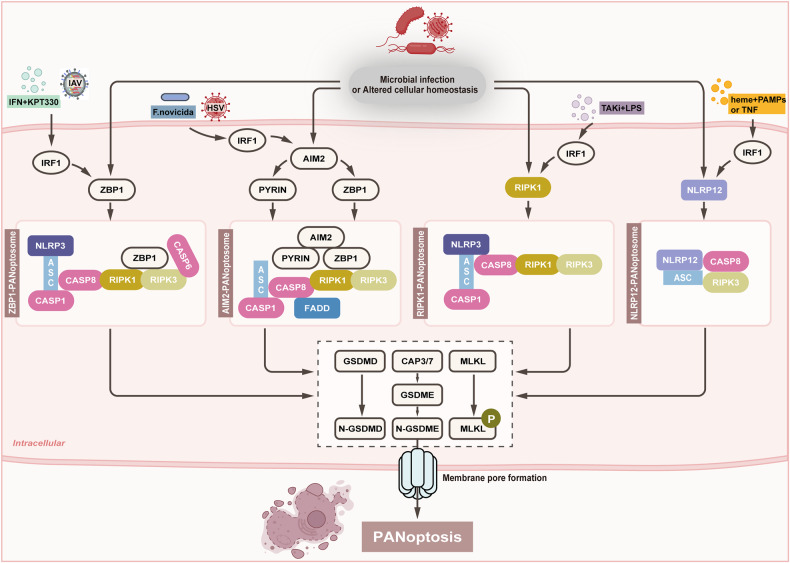
The PANoptosomes and brief molecular mechanisms of PANoptosis. When encountering triggers like diverse microbial infections and altered cellular homeostasis, sensors including ZBP1, AIM2, RIPK1, and NLRP12 can interact and recruit several other molecules to form a PANoptosome, namely ZBP1-PANoptosome (ZBP1, NLRP3, ASC, caspase-1, caspase-6, caspase-8, RIPK1, and RIPK3), AIM2-PANoptosome (AIM2, Pyrin, ZBP1, ASC, caspase-1, caspase-8, FADD, RIPK1, and RIPK3), RIPK1-PANoptosome (RIPK1, RIPK3, NLRP3, ASC, caspase-1, and caspase-8), and NLRP12- PANoptosome (NLRP12, ASC, caspase-8, and RIPK3), respectively. These PANoptosomes further induce caspase-3/7 activation, GSDMD and GSDME cleavage, and MLKL phosphorylation, resulting in membrane pore formation and PANoptosis progression. In addition, these PANoptosomes can also be regulated by IRF1 under some conditions. With IAV infection or IFN plus KPT330, IRF1 contributes to ZBP1 expression and ZBP1-PANoptosome formation. Similarly, IRF1 is responsible for AIM2-PANoptosome activation under *F. novicida* infection. IRF1 acts as an upstream regulator of RIPK1-PANoptosome with TAKi plus LPS stimulation, and IRF1 mediates the form of NLRP12-PANoptosome when stimulated with heme plus PAMPs or TNF. Abbreviations: AIM2 absent in melanoma 2, ASC apoptosis-associated speck-like protein containing a caspase recruitment domain, CASP caspase, FADD Fas-associated death domain protein, GSDMD: gasdermin D, GSDMDE gasdermin E, IAV influenza A virus, IFN interferon, IRF1 Interferon regulatory factor 1, KPT330 a nuclear export inhibitor, LPS lipopolysaccharide, MLKL mixed lineage kinase domain-like pseudokinase, NLRP3 NLR family pyrin domain containing 3, NLRP12 NLR family pyrin domain containing 12, RIPK receptor-interacting serine/threonine-protein kinase, TNF tumor necrosis factor ZBP1 Z-DNA binding protein 1.

PANoptosis involves the crosstalk and activation of different cell death molecules, which take part in the multiprotein assembly of the PANoptosome complex [6]. This specific complex is composed of numerous key pyroptotic, apoptotic, and necroptotic molecules (PANoptotic molecules) and regulates PANoptosis during altered cellular homeostasis or other unfavorable conditions, such as influenza A virus (IAV) and herpes simplex virus 1 (HSV1) infection [11]. The assembly of the PANoptosome scaffold is similar to that of inflammasomes, which are multimeric signaling complexes associated with pyroptosis. Although the composition of PANoptosomes in different studies is not entirely the same and the phenotypic members are mainly dependent on the stimulus provided, a classical PANoptosome is mainly composed of three kinds of proteins: (1) sensor proteins such as Z-DNA-binding protein 1 (ZBP1), which is also known as DNA-dependent activator of interferon regulatory factor (DAI), and NLR family pyrin domain containing 3 (NLRP3); (2) adapter proteins with a caspase recruit domain, such as apoptosis-associated speck-like protein (ASC); and (3) RIPK1, RIPK3, and caspase-1/8, which have catalytic effects [12]. To date, two classical kinds of PANoptosome complexes have been identified, including the ZBP1- and the PYHIN family member absent in melanoma 2 (AIM2)-PANoptosome [6, 7]. Depending on the upstream sensors, the ZBP1- and AIM2-PANoptosome complexes have been characterized in detail at the molecular level, and single-cell analysis of PANoptosome complexes has shown the presence of PANoptosomes under certain conditions [11]. In addition, the RIPK1-PANoptosome and NLRP12-PANoptosome also have been reported [13, 14]. Figure 1 shows these four types of PANoptosomes and brief molecular mechanisms of PANoptosis.

The ZBP1-PANoptosome mainly consists of ZBP1, NLRP3, ASC, caspase-8, caspase-1, RIPK1, and RIPK3 [6, 15]. Furthermore, recent studies have indicated that caspase-6 binds with RIPK3 to promote the crosstalk between ZBP1 and RIPK3 and to facilitate ZBP1-PANoptosome formation [16]. As a cytosolic DNA sensor, ZBP1 is a potent activator of the immune response, inducing interferon (IFN)-signaling activation and nuclear transcription factor kappa B (NF-κB) activation [17–21]. ZBP1 has been reported to function as a central regulator of inflammatory responses and cell death through the RIPK1-RIPK3-caspase-8 pathway, and ZBP1 can regulate NLRP3 inflammasome activation and trigger pyroptosis, apoptosis, necroptosis, and PANoptosis [22, 23]. In addition, after NLRP3 and caspase-8 are activated, pyroptosome ASC can be recruited and promote self-activation of inflammatory caspase-1 [22, 23], facilitating pyroptosis and interleukin-1β (IL-1β) secretion and promoting the amplification of inflammation. PANoptosomes activation and PANoptosis progression can be positively regulated by IFN regulatory factor 1 (IRF1), a member of the IRF family, and it has been reported that IRF1 can mediate PANoptosis with TNF plus IFN-γ stimulus [24, 25]. Moreover, IRF1 is a positive regulatory factor for ZBP1 [26], and it positively regulates ZBP1 expression during virus infections [27]. IRF1 contributes to ZBP1-mediated PANoptosis in response to stimulation with IFN-γ plus the nuclear export inhibitor KPT330 and IAV infection [28]. Moreover, upon IAV infection, DAI/sperm-associated antigen 9/c-Jun N-terminal kinase (DAI/SPAG9/JNK) signaling pathway can enhance the interactions among RIPK1, RIPK3, and DAI, further promoting PANoptosome formation [29].

Recently, the AIM2-PANoptosome has been identified as a PANoptosome that contains AIM2, Pyrin, ZBP1, ASC, caspase-1, caspase-8, RIPK1, RIPK3, and FADD and drives inflammatory cell death [7]. An N-terminal pyrin domain and a C-terminal oligonucleotide-binding HIN domain make up AIM2, which is a DNA sensor. The activation of AIM2 further recruits ASC and caspase-1 [30]. Furthermore, AIM2 inhibition can completely impair inflammatory cell death, and the loss of AIM2 decreases the expression of Pyrin and ZBP1, which indicates that AIM2 serves as an upstream regulator of Pyrin and ZBP1 [7]. Pyrin is a distinct sensor that specifically shares the PYD domain with NLR and PYHIN family proteins, such as NLRP3 and AIM2. Kanneganti’s team found that the loss of Pyrin induced a partial reduction in PANoptosis [7]. FADD is the adaptor for caspase-8 and is important for caspase-8-dependent inflammatory responses via the RIPK1-caspase-8-FADD complex [31]. However, recent research has also revealed that caspase-8 and FADD can inhibit spontaneous necroptotic cell death by inhibiting the expression of ZBP1 and necroptosis [32]. FADD suppresses downstream inflammation mediated by ZBP1 and TNFR1 by inhibiting caspase-8-GSDMD-dependent pyroptosis and MLKL-induced necroptosis in epithelial cells [33]. In addition, caspase-8, which is involved in inflammasome activation, can also act independently of FADD in some conditions [31]. These studies indicate that PANoptosome assembly is complex and remains confusing. In addition to ZBP1, IRF1 is also responsible for AIM2 activation and cell death under *Francisella novicida* (*F. novicida*) infection [34]. Bone-marrow derived macrophages (BMDMs) with IRF1 deficiency presented reduced cell death as well as reduced AIM2-mediated PANoptotic molecules activation in response to *F. novicida* or HSV1 infection [28].

RIPK1, a master regulator of TNFR1 signaling, promotes transcription of inflammatory cytokines and is important for cell death regulation [35]. A recent study revealed that *Yersinia* infection induces PANoptosis activation in macrophages, combined with the formation of a RIPK1-PANoptosome complex, which includes RIPK1, RIPK3, caspase-8, NLRP3, ASC, and caspase-1 [13]. Moreover, IRF1 has been identified as an upstream regulator of RIPK1-PANoptosome with transforming growth factor-β (TGF-β)-activated kinase 1 (TAK1) inhibitor ((TAKi) plus lipopolysaccharide (LPS) stimulation [28]. Recently, Kanneganti et al. also identified a NLRP12-PANoptosome component containing NLRP12, ASC, caspase-8, and RIPK3, and they illustrated that NLRP12 is important for heme plus pathogen-associated molecular patterns (PAMPs)-induced inflammasome and PANoptosome activation to drive PANoptosis [14]. In addition, when stimulated with heme plus PAMPs or TNF, IRF1 can mediate the form of NLRP12-PANoptosome in BMDMs [14, 28], illustrating that IRF1 is critical for regulating PANoptosis through its effects on the transcription and form of key PANoptosome molecules. NLRP12 is significant for normal functions and is involved in multiple diseases. For example, during *Yersinia* infection, NLRP12 acts as an inflammasome sensor to recognize *Yersinia* and promotes proinflammatory activities to resistance against bacteria [36]. NLRP12 also presents anti-inflammatory role in colon inflammation and a tumor inhibitory effect on colorectal cancer (CRC) [37, 38]. It is worth noting that other possible sensor-specific PANoptosomes that have not yet been identified can form in response to external stimuli and altered cellular homeostasis. The detailed roles and functions of these PANoptosomes in PANoptosis need further exploration.

## PANoptosis in diseases

**Fig. 2:**
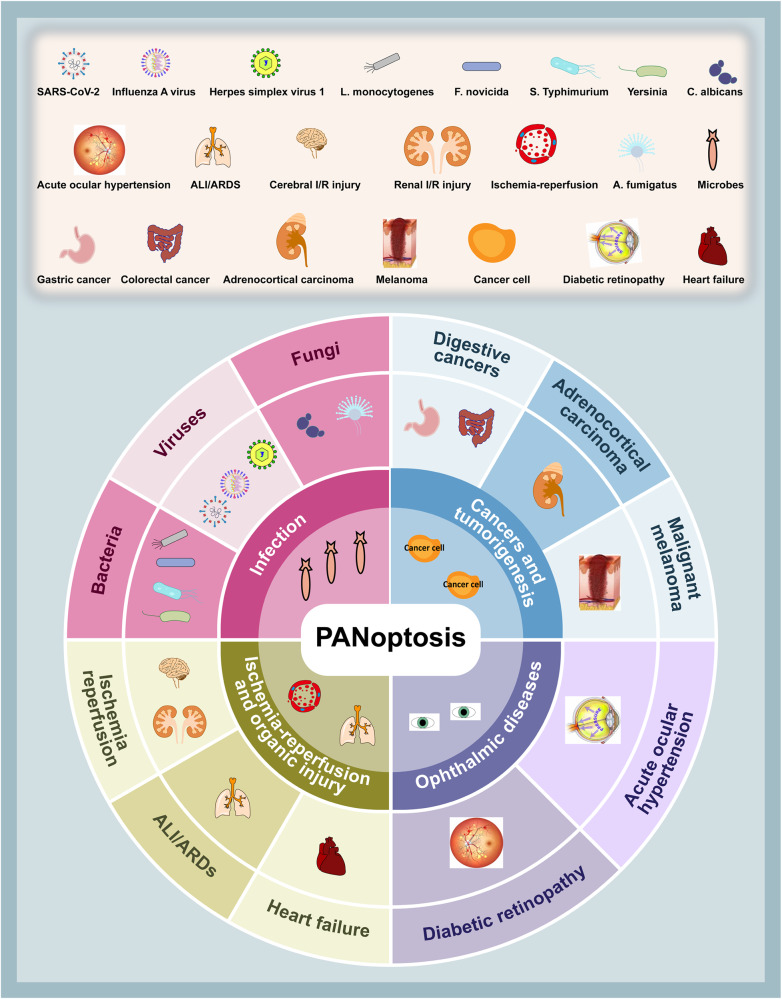
The overview of PANoptosis in diseases. PANoptosis is involved in diverse conditions, mainly including diverse infections (e.g. bacteria, viruses, fungi), cancers and tumorigenesis (e.g. digestive cancers, adrenocortical carcinoma, malignant melanoma), ischemia-reperfusion and organic injury (e.g. ALI/ARDS and heart failure), and ophthalmic diseases (e.g. diabetic retinopathy and acute ocular hypertension).

The molecular mechanisms underlying PANoptosis remain largely unknown and are complicated, involving a variety of signaling pathways. The understanding of PANoptosis in disease has grown primarily out of different pathological processes and disease classes, including infections, tumors and cancers (Fig. 2).

### PANoptosis in infections and infectious diseases

#### PANoptosis in SARS-CoV-2 infection and Coronavirus disease 2019 (COVID-19)

COVID-19 is caused by the virus SARS-CoV-2 and is mainly characterized by symptoms such as fever, cough, fatigue, shortness of breath, muscle ache, and pneumonia. This ongoing global pandemic has a negative impact on healthcare systems at all levels and several vaccines, antiviral drugs, and therapeutic antibodies are used for COVID-19 treatment [39–42]. It has been reported that SARS-CoV-2 can infect respiratory epithelial cells, as well as other immune cells [43]. Pattern-recognition receptors (PRRs), such as TLRs, retinoic acid-inducible gene I (RIG-I)-like receptors (RLRs), NLRs, and AIM2, can recognize SARS-CoV-2 RNA following infection, followed by the production of proinflammatory cytokines. Typically, imbalanced proinflammatory cytokines and cytokine storms induce inflammatory cell death, causing harmful systemic hyperinflammation and damage to multiple tissues and driving the development of COVID-19 [44, 45]. The Janus kinase/signal transducer and activator of transcription 1 (JAK/STAT1)/IRF1 signaling pathway is responsible for the induction of caspase-8-FADD-associated PANoptosis in response to the cytokines TNF-α and IFN-γ. This pathway is subsequently followed by the production of nitric oxide [25]. Furthermore, in vivo studies showed that symptoms of TNF-α and IFN-γ cotreatment were similar to severe SARS-CoV-2 patients, and treatment with TNF-α and IFN-γ neutralizing antibodies effectively protected mice against SARS-CoV-2 [25]. In addition, increased levels of ZBP1 were found in patients who succumbed to COVID-19 compared with those who recovered [18]. Moreover, ZBP1-induced PANoptosis and cytokine storms impair the treatment of COVID-19 [18]. These findings indicate that inhibiting PANoptosis may be a potential target for COVID-19.

#### PANoptosis in other microbial infections

Kanneganti et al. reported the viral triggers and bacterial pathogens like IAV, vesicular stomatitis virus (VSV), HSV1, *Listeria monocytogenes* (*L. monocytogenes*), *Salmonella enterica serovar Typhimurium* (*S.Typhimurium*), and *F. novicida* can infect macrophages and induce cell death and PANoptosis activation [6, 7, 18]. Following IAV infection, ZBP1 acts as the upstream sensor to form the ZBP1-PANoptosome and promote cell death characterized by the classical hallmarks of pyroptosis, apoptosis, and necroptosis [46]. The loss of the Zα2 domain in ZBP1 inhibits IAV-induced PANoptosis and activation of the NLRP3 inflammasome [47]. IRF1 serves as a transcriptional regulator of ZBP1, facilitating NLRP3 inflammasome activation and cell death in response to IAV infection [27]. AIM2 regulates Pyrin and ZBP1, and forms AIM2-PANoptosome, which activates inflammatory signaling and PANoptosis for host protection with HSV1 or *F. novicida* infection [7].

A recent study revealed that pathogenic *Yersinia*, which causes fatal sepsis and gastritis in humans [48], induces the formation of a RIPK1-PANoptosome complex to regulate PANoptosis [13]. Moreover, after *Yersinia* infection, YopJ is secreted into macrophages and suppresses the host protein TAK1. In TAK1-deficient cells, innate immune can cause RIPK1-independent inflammasome activation and PANoptosis [49]. In macrophages, gene and protein expressions related to PANoptosis when infected with *Enterococcus faecalis (E. faecalis)*, a bacteria commonly found in the digestive system. These expressions caused a cascade of pyroptosis, necroptosis, and apoptosis to occur [50]. In addition, it was reported BMDMs that lacking key molecules required for PANoptosis were significantly more resistant to cell death induced by *Burkholderia thailandensis* (*B. thailandensis*) [51]. PANoptosis was observed to be activated in rats with sepsis-associated encephalopathy (SAE), and the downregulation of TLR9 was found to inhibit PANoptosis, resulting in improved survival rates and reduced pathological changes in SAE rats [52]. *Candida albicans* (*C. albicans*) and *Aspergillus fumigatus* (*A. fumigatus*) induce PCD in macrophages and cytokines via ZBP1-mediated PANoptosis, which is important for controlling infections [53]. Although sensor mechanism of ZBP1 to fungi remain unclear, it is thought that fungal pathogens can produce different PAMPs, which may serve as ZBP1 ligands to drive PANoptosis [54].

In conclusion, PANoptosis seems to be a double-edged sword during different microbial infections. For example, it may be useful to inhibit PANoptosis during COVID-19 and SAE treatment, while AIM2-induced PANoptosis may provide host protection in response to HSV1 or *F. novicida* infection [7, 18, 52]. The relationships between PANoptosis and microbial infections are complex and unclear. More research is needed to examine the role of PANoptosis in these infections.

### PANoptosis in cancers and tumorigenesis

PANoptosis play a vital role in cancer biology, especially in cancer immunity and the tumor microenvironment [55]. Although cancer is characterized by resistance to PCD, but the connection between anticancer immunity and PANoptosis remains unclear. When one or more PCD pathways in cancer cells are inhibited, PANoptosis may help the host activate additional cell death defense mechanisms, which may play a protective role through cancer suppression [24]. To date, PANoptosis has been reported to play roles in several types of cancers, and the role of PANoptosis has mainly been assessed in vitro and in murine models.

Gene expression pattern in PANoptosis and identified three distinct PANoptosis profiles in individuals with gastric cancer (GC). These profiles were characterized by a significant decrease in the expression of immune checkpoint molecules [56]. CRC is a common adult cancer with a high death rate, and it remains a major public health burden worldwide [57]. Genes associated with PANoptosis, such as ZBP1, AIM2, and NLRP3, were dysregulated expression in colon cancer tissues [58]. PANoptosis was implicated in CRC and that the progression of PANoptosis inhibited IRF1-regulated tumor development in a CRC mouse model [8]. Moreover, PANoptosis induced by TNF-α and IFN-γ has been reported to block tumorigenesis in different cancer lineages, particularly in tumorigenesis associated with CRC colitis [24]. IRF1 is a transcription factor that drives caspase-mediated extrinsic or intrinsic apoptosis in multiple cancer models [59]. In WT mice, the level of expression of IRF1 is lower in colons with tumor development than in non-tumor colons [8]. IRF1 is an upstream regulator of PANoptosis that drives cell death during colitis-associated tumorigenesis and promotes PANoptosis to prevent CRC [59]. In addition, the overexpression of cysteine desulfurase (NFS1) was observed in CRC tissues [60]. The loss of NFS1 combined with oxaliplatin treatment caused PANoptosis by enhancing the intracellular expression levels of reactive oxygen species (ROS) [60], indicating that NFS1 is a negative regulator of PANoptosis in CRC.

Adenosine deaminase acting on RNA 1 (ADAR1) is a predominant inhibitor of immune responses to activate host double-stranded RNA (dsRNA) sensors [61]. Karki revealed that ADAR1 could interact with the Zα2 domain of ZBP1 to inhibit the combinations between ZBP1 and RIPK3, resulting in the inhibition of ZBP1-mediated PANoptosis and promotion of tumorigenesis in CRC [62]. A study explored the regulatory roles of long noncoding RNAs (lncRNAs) associated with metastasis and PANoptosis in colon adenocarcinoma (COAD), and revealed that lncRNA small nucleolar RNA hostgene 7 (SNHG7) was a PANoptosis-related biomarker associated with metastasis, chemoresistance, and prognosis [63]. However, how SNHG7 influences PANoptosis in COAD remains unclear and requires further research.

The cyclin-dependent kinase 1 (CDK1) is identified as a prognostic indicator of adrenocortical carcinoma (ACC) and facilitates the proliferation of ACC cells [64]. Furthermore, a study showed that cucurbitacin E (CurE), the CDK1 inhibitor, could trigger PANoptosis by regulating the interaction between CDK1 and ZBP1-PANoptosome in ACC cells [64]. Promoting PANoptosis showed good potential in preventing malignant melanoma. The combination of IFN-γ and the nuclear export inhibitor KPT330, significantly repressed melanoma by inducing ZBP1- and Zα2 domain-dependent PANoptosis [62]. In addition, metformin and doxorubicin could effectively accumulate in xenograft melanoma tumors via folic acid nanoparticles (FCA-NPs), facilitating PANoptosis in tumor cells [65]. Furthermore, a systematic framework was established to analyze PANoptosis-related biomarkers based on 32 types of pancancer data from the Cancer Genome Atlas (TCGA) database [66]. A total of 27 genes were part of the PANoptosis signature, and high expression levels of PANoptosis genes were unfavorable in multiple cancer subtypes [66]. These results indicate that PANoptosis is relatively complex in cancers, and the role of PANoptosis may be a double-edged sword in different cancers and other diseases. More research is needed to further examine the association between PANoptosis and cancers.

### PANoptosis in acute lung injury and acute respiratory distress syndrome

Acute lung injury (ALI) is caused by different kinds of lung injuries, and often induces significant morbidity, and the most serious manifestation is called acute respiratory distress syndrome (ARDS) [67, 68]. ALI/ARDS involves complement activation that may be caused by multiple inflammatory insults, including sepsis, pneumonia, COVID‐19, and traumatic injury [67, 69]. Stimulator of IFN genes (STING) is critical in host immune responses and prevents tumors and different infections, especially viral infections [70, 71]. The STING agonist diamidobenzimidazole (diABZI) caused dsDNA release, PANoptosis and NETosis and induced inflammation and ARDS [72]. Moreover, microRNA-29a-3p (miR-29a-3p) agomir therapy reduced the production of several inflammatory factors in the lungs and reduced PANoptosis by inhibiting the ZBP1-PANoptosome in alveolar epithelial cells, ultimately reversing lung injury in ALI model mice [73].

### PANoptosis in ischemia-reperfusion and organ failure

Ischemia-reperfusion (I/R) damage is a complex pathologic process that contributes to the pathological states of numerous diseases, which are mainly associated with cellular damage and death and negatively impact prognosis [74–77]. Bioinformatic analysis revealed that Bax, ZBP1, and Pycard may be involved in the crosstalk among different PCD pathways and may play important roles in PANoptosis in cerebral I/R injury [78]. Similarly, PANoptosome-associated proteins such as NLRP3, ASC, caspase-1/8, and RIPK1 were observed in cell and animal experiments of ischemic brain injury [79]. RNA-binding protein (RBP)-related genes were correlated with immune cell infiltration and PANoptosis in heart failure [80]. PANoptosis-like cell death involving important members of the PANoptosome (caspase-1, NLRP3, and RIPK3) occurred in I/R-injured retinal neurons in vitro and in vivo [81]. Furthermore, 3,4-methylenedioxy-β-nitrostyrene (MNS) significantly reduced PANoptosis through the specific inhibition of the PANoptosome protein NLRP3, protecting the kidney against renal I/R injury [82]. Taken together, these studies provided a new perspective that PANoptosis may be involved in the progression of organic I/R injury and organ failure.

### PANoptosis in ophthalmic diseases

Diabetic retinopathy is a main cause of disrupted vision and preventable blindness worldwide [83]. Dickkopf-1 (DKK1), an inhibitor of canonical Wnt signaling, can inhibit retinal neovascularization and acellular vessels and markedly ameliorate PANoptosis in retinal tissues of diabetic retinopathy rats [84]. Glaucoma is a common neurodegenerative disorder with progressive loss of retinal ganglion cells (RGCs) and defects of visual field. Dynamin-related protein 1 (Drp1) can cause the mitochondrial ROS production during glaucomatous development, blocking the balance of the redox system and inducing PANoptosis in glaucoma mice model [85]. Acute ocular hypertension (AOH), a significant manifestation of acute glaucoma, can induce retinal ischemia and death in RGCs. Melatonin was found to effectively decrease RGC loss and inhibit the upregulation of cleaved caspase-3/8, Bax, and Bcl2-associated agonist of cell death after AOH injure. Moreover, melatonin plays a neuroprotective role due by inhibiting PANoptosis in AOH retinas [86].

## Endogenous molecules and compounds that target PANoptosis

PANoptosis plays a dual role in inflammatory diseases. In addition to inhibiting inflammatory signals, PANoptosis can be induced for antitumor therapy. A potential approach is to target upstream signaling pathways, sensors, and PANoptosome components, such as ZBP1, AIM2, NLRP3, RIPK1/3 to reduce the pathological effects of inflammatory cytokines. Recently, some small molecule inhibitors targeted to PANoptosome complexes have entered clinical trial stages, while others have mainly been used in the lab and will required improvement to produce viable treatments. In addition, some studies have demonstrated that some endogenous molecules/drug candidates are relevant to the pathogenesis of multiple diseases by directly regulating PANoptosis.

### Endogenous molecules that directly regulate PANoptosis

After SARS-CoV-2 infection, cell death is characterized in the inflammatory cell by combined TNF-α and IFN-γ administration [25]. FUN14 domain containing 1 (FUNDC1) interacted with mitochondrial Tu translation elongation factor to prevent cytoplasmic mitochondrial DNA release and PANoptosome activation [87]. On the other hand, some endogenous molecules, such as ADAR1 and DKK1, have been identified as inhibitors of PANoptosis [62]. DKK1 protected streptozotocin (STZ)-induced diabetic rats by suppressing PANoptosis and retinal neovascularization [84]. Furthermore, NFS1 deficiency combined with oxaliplatin triggered PANoptosis in vivo and in vitro [60]. The information regarding the direct targeting of PANoptosis by these endogenous molecules is shown in Table 1.

**Table 1:** Endogenous molecules that regulate PANoptosis.

Endogenous molecule	Target/mechanism	Disease	References
IRF1	/	colorectal cancer	[8]
miR-29a-3p	Down-regulated TNF-α, IL-1β, and IL-6 in the lungs, reducing alveolar epithelial cell PANoptosis by the downregulation of ZBP1, GSDMD caspase-3, caspase-8, and MLKL	acute lung injury/acute respiratory distress syndrome (ALI/ARDS)	[73]
TNF-α and INF-γ	Activated the JAK/STAT1/IRF1 axis, inducing nitric oxide production and driving caspase-8/FADD-mediated PANoptosis	SARS-CoV-2	[25]
NFS1	Prevented PANoptosis in an S293 phosphorylation-dependent manner under oxaliplatin treatment	colorectal cancer	[60]
DKK1	Inhibited PANoptosis by the blockage of GSDMD, caspase-3, RIPK3	Diabetic retinopathy	[84]
ADAR1	Suppressed PANoptosis by interacting with the Zα2 domain of ZBP1 to limit ZBP1 and RIPK3 interactions	colorectal cancer and melanoma	[62]
FUNDC1	Inhibited cytoplasmic release of mitochondrial DNA and activation of PANoptosome via interaction with TUFN	DOX cardiotoxicity	[87]

### Compounds that directly regulate PANoptosis

Similarly, some compounds can directly regulate PANoptosis. Messaoud-Nacer et al. reported diABZI caused IFN-dependent acute lung inflammation, PANoptosis-mediated cell death and inflammatory cytokine production [72]. Melatonin exerted a neuroprotective effect by inhibiting PANoptosis during AOH [86]. MNS significantly protected the kidney against renal ischemia-reperfusion (RIR) damage by reducing PANoptosis by specifically inhibiting NLRP3 [82]. CurE exhibited the strongest inhibitory effect as a CDK1 inhibitor by regulating PANoptosis in ACC cells in a ZBP1-dependent manner [64]. Detailed information on these compounds is shown in Table 2.

**Table 2:** Compounds that regulate PANoptosis.

Compound	Structure	Target/mechanism	Disease	Reference
diABZI		STING agonist triggered PANoptosis	Acute respiratory distress syndrome (ARDS)	[72]
Melatonin		Inhibited PANoptosis by reducing the expression of NLRP3, ASC, cleaved caspase-1, GSDMD, and cleaved GSDMD	Acute ocular hypertension (AOH)	[86]
Cucurbitacin E		Inhibited CDK1, CDK1 regulated PANoptosis of ACC cells through binding with the PANoptosome in a ZBP1-dependent way	Adrenocortical carcinoma (ACC)	[64]
MNS		Reduced PANoptosis by inhibition of NLRP3	Renal ischemia-reperfusion Injury	[82]

### Compounds targeting the main targets of PANoptosis

#### Caspases

There were 14 members had been identified in mammal caspase family, which could be grouped into apoptotic subtypes (caspase 3, 6–10) and inflammatory subtypes (caspase 1, 4, 5, and 11) [88]. In addition to mediating apoptosis, caspase-6 can bind RIPK3 and enhance the interaction between ZBP1 and RIPK3, resulting activation of PANoptosis pathway [16]. Caspases are involved in a variety of damage-associated molecular patterns (DAMP)- or PAMP-induced cell death pathways and human diseases (Table 3). VX-765, a highly selective inhibitor of IL-converting enzyme (ICE)/caspase-1 [89], is widely used in caspases studies. Tetracycline decreased the production of IL-1 and IL-18 in alveolar leukocytes from ARDS patients and reduced pulmonary inflammation by inhibiting caspase-1-dependent cytokines production [90]. The brain-penetrable caspase-1 inhibitor CZL80 significantly reduced neuronal excitability and the incidence of Febrile seizures production in neonatal mice and alleviated the enhanced susceptibility to epileptogenesis [91, 92].

**Table 3:** Compounds that target caspases.

Compound	Structure	Target	Clinical stage	Indications	References
Tetracycline		Caspase-1	Phase IV.	Inflammatory diseases	[90]
Emricasan		Pan-caspase	Phase II-terminated	NASH cirrhosis	[177]
CZL80		Caspase-1	N/A	N/A	[91, 178]
Belnacasan (VX-765)		Caspase-1	N/A	N/A	[179, 180]
Ac-YVAD-cmk		Caspase-1	N/A	N/A	[181, 182]

#### NLRP3

NLRP3 is a tripartite protein and the most representative inflammasome sensor of the NLR family. The representative specific NLRP3 inhibitors are listed in Table 4. MCC950, one of the most effective and specific NLRP3 inhibitors, has been shown to be effective in a variety of NLRP3-dependent murine disease models [93]. MCC950 specifically inhibited the activation of NLRP3 inflammasomes in macrophages without impairing other inflammasomes. MCC950 has entered in phase II clinical trials for rheumatoid arthritis [94]. OLT1177 (dapansutrile) is a small orally available NLRP3 inhibitor that has shown initial efficacy both in vitro and in vivo [95, 96]. In a phase 1b study, dapansutrile administration was tolerated well in patients with reduced ejection fraction and stable heart failure [97]. OLT1177 inhibited the release of IL-1 and IL-18 at nanomolar concentration in vitro, and none significant effect on the NLR family caspase recruitment domain-containing 4 (NLRC4) or AIM2 inflammasomes [95].

**Table 4:** Compounds that target NLRP3.

Compound	Structure	Clinical stage	Indications	References
CP-456,773 (MCC950)		Phase II-terminated	Rheumatoid arthritis	[93, 183]
Glyburide		Phase IV	Type 2 diabetes	[98, 184]
Colchicine		Phase IV	Gout and FMF	[185]
OLT1177		Phase II	NA	[95, 97, 186]
11673-34-0		NA	NA	[101]
JC-171		NA	NA	[102]
Fenamate		NA	NA	[103]
CY-09		NA	NA	[105, 187]
BOT-4-one		NA	NA	[106, 108]
Fclla-2		NA	NA	[109]
3,4-Methylenedioxy-β-nitrostyrene		NA	NA	[110]
NBC series		NA	NA	[111]
Parthenolide		NA	NA	[112]
BAY 11-7082		NA	NA	[112, 188]
INF39		NA	NA	[115]
Spirodalesol		NA	NA	[116]
Oridonin		NA	NA	[189]

Glyburide is an insulin secretion activator and has been approved to treat type-II diabetes [98]. Glyburide can effectively block multiple NLRP3-dependent pathologies, such as bronchopulmonary dysplasia and Crohn’s disease [98, 99], by suppressing the release of proinflammatory cytokines and chemokines [100]. Glyburide does not alter the expression of NLRP3, suggesting that inhibition occurs at NLRP3 and upstream of ASC [98]. An intermediate substrate without cyclohexylurea moiety (CAS No. 16673-34-0) inhibited NLRP3­mediated myocardial injury in a mouse model and none affected glucose metabolism [101]. JC­171, the hydroxysulfonamide glyburide analog [102], inhibited J774A-induced IL-1 release in the presence of LPS/adenosine triphosphate (ATP) by interfering with LPS- and ATP-induced NLRP3/ASC interactions. The fenamate class of nonsteroidal anti-inflammatory drugs (NSAIDs) can inhibit NLRP3 in macrophages by inhibiting the volume-regulated anion channel, which is independent of cyclooxygenase enzymes [103, 104]. In response to ATP, nigericin, and monosodium urate, fenamates specifically and reversibly inhibit NLRP3 stimulation without affecting AIM2 or NLRC4 activation.

Structure-activity relationship studies of C172 identified that CY-09 directly binds to the ATP-binding motif of the NLRP3 NACHT domain [105]. In addition, CY-09 is active in patient’s synovial fluid cells or monocytes from healthy individuals. BOT-4-one is a well-known covalent modifier and inhibits T cell receptor-mediated NF-κB signaling and JAK/STAT3 signaling [106, 107]. BOT-4-one alkylation of NLRP3 impaired ATPase activity, slowed the assembly of the NLRP3 inflammasome, and increased NLRP3 ubiquitination [108]. A screened inhibitor Fc11a-2, which treated dextran sulfate sodium (DSS)-induced murine colitis by targeting the NLRP3 inflammasome [109]. He et al. screened a kinase inhibitor library and revealed MNS as a potential NLRP3 inhibitor without affecting NLRC4 or AIM2 inflammasome activation or K^+^ efflux induced by NLRP3 agonists [110].

The 2-Aminoethoxy diphenylborate (2APB) is a boron-based calcium channel inhibitor that inhibits NLRP3 and cellular Ca2^+^ homeostasis. Baldwin and colleagues developed a series of novel boron compound (NBC) inhibited the NLRP3 inflammasome and functioned independently of Ca2^+^ [111]. Parthenolide and Bay11-7082 also inhibited NLRP3 ATPase activity in vitro [112], but are unlikely to act as specific NLRP3 inhibitors due to their diverse biological activities. Some acrylamide derivatives were developed to lower the electrophilicity of the agent to increase safety and selectivity. Compound 9 and other structurally related compounds inhibited the activities of NLRP3 ATPase and caspase-1, as demonstrated by their initial design of a series of unsaturated esters [113]. INF58 was able to concentration-dependently inhibit NLRP3 ATPase as one of the most potent analogs [114]. And an irreversible NLRP3 inhibitor known as Compound 11 (INF39) was able to inhibit caspase-1 but not IL-1 release from macrophages [115]. Spirodalesol is a racemic mixture that was isolated from *Daldinia eschscholtzii*., it prevented macrophages secreted IL-1 by inhibiting NLRP3 inflammasome assembly [116].

#### RIPK1

RIPK1 is a master regulator of the cellular fate between the pro-survival and death signaling pathways [117]. The development of highly selective RIPK1 inhibitors focuses on the unique hydrophobic pocket in the allosteric regulatory domain [118]. The RIPK1 inhibitors can be roughly divided into three categories, the DFG-in conformation of RIP1 is the target of type I RIPK1 inhibitors, whereas the inactive DFG-out conformation of RIP1 is the target of type II and III RIPK1 inhibitors at distinct sites [119, 120]. Tozasertib (VX-680) and sunitinib are two examples of Type I RIPK1 inhibitors that were identified by screening collections of kinase inhibitors. However, these inhibitors typically exhibit low specificity. A series of tozasertib analogs have been developed that had higher selectivity for RIPK1 but with lower potencies [121]. In general, type III inhibitors have a higher kinase selectivity than that of type I and II inhibitors (see Table 5), and some of them have entered clinical trials. Screening of the necrostatin derivatives led to novel RIPK1 inhibitors with potential for development [122, 123]. For example, Nec-5 exhibited more efficacy than Nec-1 in preventing death in FADD-deficient Jurkat cells [124]. Nec-21 was shown to be a dual inhibitor of RIPK1 and JNK3 in a preclinical study [125]. The Nec-1 analog DIMO protected heart from I/R damage [126]. The combination of ponatinib and necrostatin-1 created a highly powerful and selective hybrid RIPK1 inhibitor PN10 [127]. GSK963 and its analogues with a dihydropyrazole scaffold were more effective than Nec-1 in vitro and contributes to our understanding of the function of RIPK1 in disease etiology [128]. The Phase 1 study on the combination of GSK095 and a checkpoint inhibitor was started for pancreatic ductal adenocarcinoma since it has been hypothesized that RIPK1 kinase encourages macrophage-mediated adaptive immunological tolerance in pancreatic cancer [129]. GSK481 was found by the screening of DNA-encoded small-molecule libraries (DEL) of GSK against RIPK1 [130], which was further optimized to GSK772. There were over ten clinical studies have been conducted to evaluate GSK772, the first RIPK1 inhibitor licensed for clinical investigation was optimized using DEL technology since 2014 [120, 131, 132]. GSK772 has been in phase II clinical studies for plaque-type psoriasis, ulcerative colitis, and rheumatoid arthritis [133–137]. GSK772 suppresses multiple types of inflammation, however, moderate brain permeability has limited its use in neurodegenerative disease [138].

**Table 5:** Compounds that target RIPK1.

Compound	Structure	Clinical stage	Indications	References
GSK963		N/A	N/A	[128]
GSK095		Phase I/II-terminated	Pancreatic cancer	[129]
GSK547		NA	NA	[190]
GSK481		NA	NA	[130]
GSK772		Phase II-completed	Ulcerative colitis; arthritis; psoriasis	[134, 191, 192]
DNL104	Unpublished	Phase Ia -terminated	NA	NA
DNL747	Unpublished	phase Ib/IIa-terminated	Amyotrophic lateral sclerosis; Alzheimer disease	[143]
DNL788	Unpublished	Phase II	Amyotrophic lateral sclerosis; Multiple sclerosis	NA
DNL758	Unpublished	Phase II	Cutaneous lupus erythematosus; ulcerative colitis	NA
SIR1-365	Unpublished	Phase I	SIRS, corona virus infection	NA
ABBV-668	Unpublished	Phase II	Ulcerative colitis	NA
R552	Unpublished	Phase II	Peripheral autoimmune	NA
Necrostatin-1s (Nec-1s)		NA	NA	[193, 194]
Necrostatin-5		NA	NA	[124]
Necrostatin-21		NA	NA	[125]
DIMO		NA	NA	[126]
PN10		NA	NA	[127]
Compound 21 (ZB-R-55)		NA	NA	[139]
PK68		NA	NA	[140]
Compound 70		N/A	NA	[141]
GNE684		NA	NA	[145, 146]
Zharp1-211		NA	NA	[148]
SZM679		NA	NA	[149]
RIPA-56		N/A	N/A	[151, 195]
Nigratine (6E11)		NA	NA	[153, 196]
AV123		NA	NA	[155]
MBM105		NA	NA	[155]
Compound 20		NA	NA	[156]
Compound 8		N/A	N/A	[193]
Compound 22		NA	NA	[197]
Butylate hydroxyanisole (BHA)		NA	NA	[198]

The treatment of systemic inflammatory response syndrome (SIRS) and sepsis is interesting given the molecular function of RIPK1 in sepsis. ZB-R-55 was approximately ten times more potent than GSK772 and exhibited excellent kinase selectivity, good oral pharmacokinetics, and good therapeutic effects in an LPS-induced sepsis model [139]. Phase I clinical study to evaluate the safety and efficacy in patients with severe COVID-19 was completed in 2021. After that, a phase I clinical study was started to examine the safety, pharmacokinetics (PK), and pharmacodynamics (PD) in SIRS patients. Furthermore, ABBV-668 is an investigational drug in phase II clinical development for the treatment of moderate to severe ulcerative colitis. Hu and colleagues discovered PK6 and its derivatives as a novel class of inhibitors of necroptosis that directly inhibit RIPK1 kinase activity [140]. The researchers then made Compound 70, an analog of PK6, which had much better metabolic stability than PK68 in human and rat liver microsomes [141].

DNL104 was the first brain permeable RIPK1 inhibitor that entered clinical trial stage [142]. However, the clinical study demonstrates that DNL104 inhibits RIPK1 but is not associated with toxicities to the central nervous system. Denali Therapeutics began a phase Ia clinical trial for SAR443060 (DNL747), a small-molecule, selective, orally bioavailable, central nervous system (CNS)-penetrant, reversible RIPK1 inhibitor, phase Ib/IIa trials in amyotrophic lateral sclerosis (ALS) and Alzheimer’s disease (AD) followed [143]. Denali Therapeutics recently announced that a CNS-penetrant backup compound for DNL747, SAR443820 (DNL788), successfully completed first in human studies and began a phase II study in patients with ALS in 2022. The first RIPK1 inhibitor to be approved for use in a clinical trial in China, GFH312, has completed a phase I clinical trial to test its safety, tolerability, and PK in healthy subjects. The data from completed phase I trial in Australia demonstrated excellent safety profile and desirable PK & PD properties of GFH312 [144]. GFH312 has now recruited for Phase II clinical trials in patients with peripheral artery disease and with intermittent claudication.

As a tool compound, GNE684 has been extensively used in mechanistic studies and animal models of human diseases to investigate the function of RIPK1 [145–147]. Zharp1-211, a selective and potent RIPK1 inhibitor that restores intestinal homeostasis, significantly reduces JAK/STAT1-mediated expression of chemokines and MHC class II molecules in intestinal epithelial cells [148]. SZM679, a fluorine-substituted benzothiazole derivative was reported as RIPKs inhibitor and exhibited significant RIPK1 selectivity [149]. High-throughput screening (HTS) identified the benzoxazepine amide compound SN-6109, which had good RIPK1 inhibitory activity and druglike properties [150]. RIPA-56 protects organ damage against TNF-induced lethal shock by targeting RIPK1 in humans and mice [151]. SZ-15, an interesting high-molecular-weight RIPK1 inhibitor, alleviated DSS-induced UC and suppressed proinflammatory cytokines in vivo [152].

Natural product 6E11 is an extremely selective RIPK1 inhibitor that prevents necroptosis induced by TNF-α or TNF-related apoptosis-inducing ligand (TRAIL) and protects cells from injury caused by cold hypoxia or reoxygenation [153]. Benchekroun and colleagues discovered that a simplified benzazole moiety (benzosceptrin B) inhibited RIPK1 [154]. They then obtained new benzosceptrin B-derived necroptosis inhibitors with improved PIPK1 kinase inhibitory activity (AV123 and MBM105) [155]. Wang et al. reported an oleanolic acid base necroptosis inhibitor CDDO and its analogs prevented necrosome formation by targeting Hsp90 to inhibit RIPK1 and RIPK3 phosphorylation in necroptotic cells, and reduced TNF-induced SIRS as well as cerebral I/R injury in vivo [156].

#### RIPK3

RIPK3 is composed of a RIP homotypic interaction motif and plays a role in the physiological response to infection, inflammation, and cell stress. To date, most RIPK3 inhibitors are type II kinase inhibitors (Table 6). The first RIPK3 inhibitor family was reported by GlaxoSmithKline in 2013 [157, 158]. However, the high cytotoxicity of these compounds limited their development as anti-inflammatory agents. AZD5432 was discovered as a novel RIPK3 inhibitor for acute kidney injury (AKI) treatment by using virtual screening method [159]. AZD5423 significantly inhibited RIPK3 activation and MLKL phosphorylation in response to cisplatin, H/R, and TNF stimulation as well as the chemotherapy-induced AKI in a mouse model. CPD42 prevented the formation of RIPK1-RIPK3 necrosome [160], which by specifically targeting and inhibiting RIPK3-mediated necroptosis [161]. HS-1371 is a potent RIPK3 inhibitor that binds directly to RIPK3 in a time-independent and ATP-competitive manner [162]. Rodriguez et al. reported that GW39B could specifically prevent necroptosis in a panel of cell lines, but not in NIH-3T3 cells because RIPK3 did not have the RIP homotypic interaction motif (RHIM) domain [163].

**Table 6:** Compounds that target RIPK3.

Compound	Structure	Clinical stage	Indications	References
GSK840		NA	NA	[158]
GSK843		NA	NA	[158]
GSK872		NA	NA	[158]
AZD5423		NA	NA	[159]
CPD42		NA	NA	[161]
HS-1371		NA	NA	[162]
GW39B		NA	NA	[163]

### Compounds target other molecules of PANoptosis

There were some molecules targeted to other PANoptosis-related proteins, such as ZBP1, AIM2, ASC, and pore-forming molecules GSDMD and MLKL (Table 7). ZBP1 recruits RIPK3 and initiates necroptosis when Z-form RNA accumulates in the early stages of vaccinia virus (VACV) infection without the E3 domain [164]. ZBP1 also prevented ADAR1 from inhibiting Z-RNA-dependent endogenous activation of pathogenic type I IFN responses [21]. Furthermore, Reuver et al. found that ADAR1’s Z domain mediated A-to-I editing of endogenous Alu elements, preventing the pairing of inverted Alu repeats that could otherwise cause ZBP1 to be activated by dsRNA [165]. The small-molecule curaxin CBL0137 was found to reverse immune checkpoint blockade unresponsiveness in mouse models of melanoma, induce ZBP1-dependent necropsy in cancer-associated fibroblasts, and potently activate ZBP1 by triggering Z-DNA formation [166]. IFI16 shares a cytoplasmic location with AIM2, inhibits the formation of a functional AIM2-ASC complex by interacting with AIM2 and isolating cytoplasmic dsDNA because of human IFI16 lacks the pyrin domain, rendering it inaccessible to AIM2 sensing [167]. Compound 8 A is a good selective inhibitor of NLRP3 inflammasome assembly [168]. However, it only inhibited ASC oligomerization rather than the inflammasome priming phase.

**Table 7:** Compounds that target other PANoptosis-related molecules.

Compound	Structure	Target	Clinical stage	Indications	References
CBL0137		ZBP1	NA	NA	[166]
J114		NLRP3/AIM2	NA	NA	[199]
Obovatol		NLRP3/AIM2	NA	NA	[200]
Compound 8 A		ASC	NA	NA	[168]
LDC7559		GSDMD	NA	NA	[169]
Disulfiram		GSDMD	Phase IV	Alcohol dependence	[171]
Necrosulfonamide		MLKL/GSDMD	NA	NA	[173–175]
Dimethyl fumarate		GSDMD	Phase IV	Relapsing multiple sclerosis	[201]

Moreover, Sollberger et al. identified that LDC7559 (a pyrazolo-oxazepine scaffold–based molecule) as an inhibitor of GSDMD, which binds GSDMD, blocks the activity of the GSDMD N terminus and inhibits inflammasome activation [169]. Disulfiram is an FDA-approved drug to treat chronic alcohol addiction [170]. Recent studies suggested that disulfiram inhibited pore formation by GSDMD, which blocked pyroptosis and cytokine release in cells and LPS-induced septic death in mice [171]. Furthermore, administration of dimethyl fumarate reacted with GSDMD to form S-(2-succinyl)-cysteine at key cysteine residues and that succinylation of GSDMD prevented it from interacting with caspases [172].

Furthermore, necrosulfonamide (NSA) was initially identified as a selective MLKL-targeted necroptosis inhibitor, which can prevent the interaction of MLKL-RIPK1-RIPK3 necrosome complex with downstream molecules, and multiple studies have shown that NSA inhibits necroptosis thus alleviating the symptom of diseases [173]. However, subsequent studies have found that NSA act as a direct chemical inhibitor of GSDMD and binds to GSDMD to inhibit GSDMD-mediated pyroptosis [174]. In addition, recent study found that NSA inhibited phosphorylated MLKL and N-GSDMD expression in dextran sodium sulfate-induced colitis mouse model, inhibited N-GSDMD expression in bone marrow-derived macrophages and phosphorylated MLKL in NCM460 cells [175]. Therefore, NSA is a dual-target pore-forming molecular inhibitor.

Activation of an apical sensor, such as ZBP1, NLRP12, AIM2, or RIPK1, triggers the formation of a PANoptosome complex. Assembly of the PANoptosome leads to the induction of three arms of cell death: pyroptotic, apoptotic, and/or necroptotic. Inhibition of PANoptosis or PANoptosis-induced inflammation can occur at multiple levels. By direct blocking sensor molecules like ZBP1, cell death can be prevented [46], while inhibition of specific executioners of one arm of cell death can steer the death process towards a different arm of PANoptosis [176]. Consequently, targeting a single pathway of cell death, without consideration the existence of other redundant pathways can result in initially promising therapeutics that ultimately fail as the molecular lines between individual cell death pathways become blurred. Current strategies to inhibit inflammatory cell death include targeting sensor molecules, such as NLRP3, ZBP1, inhibiting enzymatic targets, such as RIPK1, RIPK3, and neutralizing downstream cytokine signaling, such as IL-1 signaling. Although these inhibitors were primarily designed to target pyroptosis, apoptosis, and necroptosis, it is critical to evaluate their effectiveness in the context of PANoptosis

## Conclusions

PANoptosis is a newly discovered cell death subroutine involving several PCD processes, highlighting significant crosstalk among pyroptosis, apoptosis, and/or necroptosis. Emerging evidence indicates that PANoptosis is involved in different conditions, such as microbial infections, cancers, ALI/ARDS, I/R, and organ failure. Although the detailed mechanisms of PANoptosis in these disorders are not completely clear, the role of PANoptosis seems to be a double-edged sword in response to different stimuli. For example, PANoptosis participates in host protection against HSV1 and *F. novicida* infection and inhibits and kills cancer cells in colorectal cancer, adrenocortical carcinoma, and other cancers. However, PANoptosis mainly negatively impacts COVID-19 and ALI/ARDS. Targeting PANoptosis with small-molecule compounds or endogenous molecules could be potential therapies, but caution is needed due to the risk of unwanted cell death and immune dysfunction. Further research on PANoptosome composition and function is necessary for better understanding and treatment development.

## Data Availability

All the data supporting the findings of this study are available from the corresponding author on reasonable request.
